# Implementation of pharmaceutical services in Brazilian primary health care: a cross-sectional study

**DOI:** 10.1186/s12875-021-01516-7

**Published:** 2021-08-25

**Authors:** Nathália Cano Pereira, Vera Lucia Luiza, Mônica Rodrigues Campos, Luisa Arueira Chaves

**Affiliations:** 1 Department of Medicines Policy and Pharmaceutical Service, National School of Public Heath Sergio Arouca, Oswaldo Cruz Foundation, Rio de Janeiro, Brazil; 2 Social Science Department, National School of Public Heath Sergio Arouca, Oswaldo Cruz Foundation, Rio de Janeiro, Brazil; 3 Federal University of Rio de Janeiro, Campus Macaé, Rio de Janeiro, Brazil

**Keywords:** Implementation Fidelity, Implementation research, Pharmaceutical service, Primary health care

## Abstract

**Background:**

In the Brazilian public health system, primary health care (PHC) is provided by the municipalities and is considered the entry level of the Unified Health System (SUS). Governmental pharmaceutical services (PharmSes) are part of the SUS, including PHC, and are the most significant way in which patients access medicine and services. Considering the diversity of the country, the municipalities have the autonomy to decide how PharmSes are implemented. Even though policies and procedures should be implemented as expected by policy makers and experts, municipality characteristics may interfere with implementation fidelity. Therefore, this study evaluated the degree to which the PharmSes in PHC were delivered as intended in Brazilian municipalities.

**Methods:**

We analysed data from a secondary database originating from a cross-sectional nationwide study carried out by the Ministry of Health and the World Bank from 2013 to 2015. Data on 465 municipalities and the Federal District were collected from 4939 governmental PharmSes. A rating system comprising 43 indicators was developed and applied to the dataset to obtain the implementation degree (ID) of each PharmSe. Additionally, the IDs of the two PharmSes dimensions and the nine components were measured.

**Results:**

Overall, the ID of the PharmSes in Brazilian PHC was evaluated as critical. The ID was critical in 81% of the municipalities (*n* = 369), incipient in 14% (*n* = 65) and unsatisfactory in 4.8% (*n* = 22). Regarding the PharmSes dimensions, the ‘medicine management’ (MM) ID was considered critical (Mean = 46%), while the ‘care management’ (CM) ID was incipient (Mean = 22%). In terms of the PharmSes components, the highest ID was achieved by ‘forecasting’ (58%). In contrast, ‘continuing education and counselling’ showed the lowest figure (ID = 11%) in the whole sample, followed by ‘information and communication’ and ‘teamwork’.

**Conclusions:**

The degree to which PharmSes were implemented was critical (ID< 50%). This analysis demonstrated that PharmSes were implemented with low fidelity, which may be related to the low availability of medicine in PHC. Although the care management component requires more attention, considering their incipient ID, all components must be reviewed. Municipalities must increase their investment in PharmSes implementation in order to maximize the benefits of these services and guarantee the essential right of access to medicine.

## Background

Primary health care (PHC) must be prepared to address nearly all of the most common health conditions that arise in a population [1]. In Brazil, governmental PHC is provided by the municipalities and is considered the entry level of the Unified Health System (SUS – acronym in Portuguese). PHC teams are responsible for providing health services and coordinating patient care across various settings and health system levels [2].

Pharmaceutical services (PharmSes) are part of the health system, including PHC. They are provided free of charge in the SUS and are the most significant way in which patients access medicine and services [3]. PharmSes are defined as a set of actions in the health system aiming to guarantee continuous attention to the population’s health needs, both individually and collectively, through promoting equitable access to medicine and its adequate use. These actions must be developed by a pharmacist or under his or her supervision, always in cooperation with a PHC team, to improve people’s quality of life [4].

The Brazilian federal government published the National Medicine Policy (NMP) and the National Pharmaceutical Services Policy (NPSP) to outline what municipalities must do to guarantee access to medicine and its adequate use. In line with these policies, experts from the Brazilian Federal Council of Pharmacy (CFF, acronym in Portuguese), National Health Surveillance Agency (ANVISA, acronym in Portuguese), and Federal Public Health Department and researchers have published a range of documents, such as recommendations [5, 6], quality standards [7], papers [8–10] and manuals [11, 12], to support municipalities in implementing PharmSes. Despite municipalities’ characteristics (in terms of population, deprivation, epidemiology profile, etc.), PharmSes should be deployed as expected by specialists and in accordance with policies, official documents and the literature [13]. However, the municipalities have discretion in implementing PharmSes, as the health system is decentralized in Brazil [14]. This autonomy may interfere with implementation fidelity.

Implementation fidelity is a component of implementation research in which studies aim to promote the translation of evidence into routine practice. Fidelity refers to the degree to which interventions are delivered as intended by program developers, with adherence to guidelines or manuals. It is a key aspect of intervention implementation in community settings [15, 16]. Then, fidelity in PharmSes’ implementation at the PHC level can be a significant step to guarantee access to quality, safety, and efficacious medicine in an appropriate and timely manner. Verifying fidelity is critical for the accurate interpretation of PharmSes outcomes and effectiveness [17].

Even though the relevance of examining implementation fidelity is widely recognized, the literature suggests that it has not often been done [15, 16, 18]. Implementation studies of PharmSes have appeared in the literature in the last few years [19–22], but they have mainly focused on the development and evaluation of new programmes rather than monitoring and improving the fidelity of ongoing interventions [23]. Additionally, the approach of most of the studies published as peer reviewed papers is based on a single component of an intervention, such as clinical guidelines [24]. Thus, a deep understanding of the implementation of multicomponent interventions in community pharmacies remains a global issue [20, 25].

In Brazil, there have been efforts to improve PharmSes evaluation, and some studies can be understood as implementation research despite not explicitly naming this approach [26–29]. However, because of the country’s size and complexity, most studies are local. In this scenario, it is important to highlight the National Research on Access and Use of Medicines (PNAUM, acronym in Portuguese), composed of two approaches, a household and a PharmSe survey, through which data were collected from 2014 to 2015 [30, 31]. The PNAUM was a milestone for understanding the global picture of access to and use of medicine in the Brazilian population. Nonetheless, no study until now has clearly addressed PharmSes implementation fidelity.

## Methods

### The aim

This study aimed to evaluate the degree to which PharmSes in PHC were delivered as expected in Brazilian municipalities.

### Design and setting

We analysed data from a secondary database originating from the “Pharmaceutical Services in the Brazilian Healthcare Networks: an approach in the QualiSUS-Network regions Study”. This cross-sectional nationwide study was carried out by the Ministry of Health (MoH) and the World Bank (WB) from 2013 to 2015. The QualiSUS-Network was a strategy to support 15 priority regions in Brazil to improve healthcare management and quality of care through the Regional Healthcare Network [32]. These 15 regions included 10 metropolitan areas and five regions with unique social and geographic characteristics according to the MoH and the WB, such as a higher prevalence of neglected diseases, lower health coverage and a lower Human Development Index (HDI) [33].

Data from 465 municipalities and the Federal District, comprising 17 of the 27 Brazilian states, were collected by structured interviews with 4939 professionals responsible for the pharmacies of the following services: primary health units/health centres/health posts, hospitals, psychosocial care centres, prison systems, pharmaceutical supply centres and pharmacies in separate buildings. All SUS pharmacies in those municipalities were included. The data-collecting tools covered management, infrastructure and services delivery [8].

### Sampling

In this article, the analysis focused on 4094 pharmacies, either within a PHC facility or in separate buildings (dispensing PHC medicine).

The units of observation were the pharmacies that delivered PharmSes, and the unit of analysis was the municipality. The data were of good quality and complete, with duplicate cases and missing data accounting for less than 1.6%.

### Data analysis

**Fig. 1:**
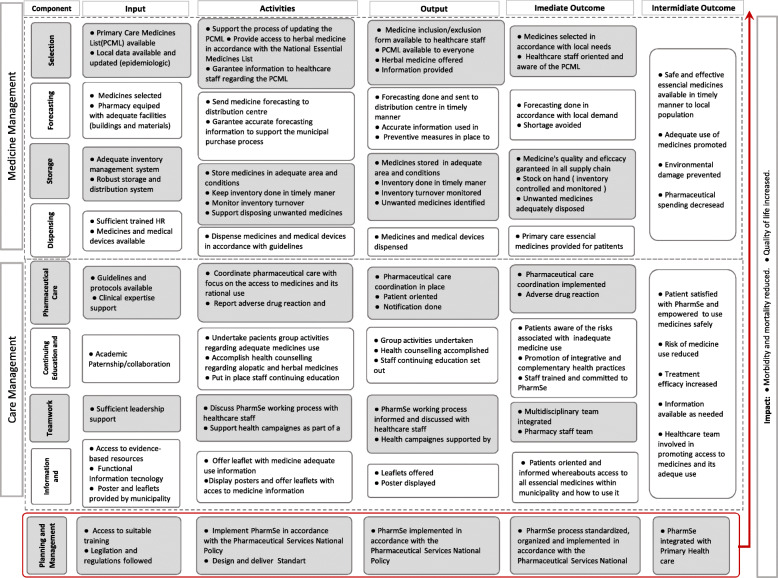
Pharmaceutical services logic model in Brazilian primary health care

In this study, the PharmSes logic model (Fig. 1) depicts the activities expected to be implemented by municipalities. PharmSes constitute a multicomponent intervention organized into two dimensions and nine components. The first dimension, ‘medicine management’, involved four components focused on medicine availability and quality: ‘selection’, ‘forecasting’, storage’ and ‘dispensing’. The second, named ‘care management,’ included four clinical components: ‘pharmaceutical care coordination’, ‘continuing education and counselling’, ‘teamwork’ and ‘information and communication’. ‘planning and management’ was considered a transversal component of these two dimensions.

A rating system comprising 43 indicators categorized into these nine PhamSes components was applied to the dataset to obtain the implementation degree (ID). All indicators had a maximum score of 10 points. The details of the logic model and rating system are described elsewhere in the literature [34].

The median of the points obtained for each indicator was used as a summary measure to define the municipality ID. The cut-off point was determined by quartiles: incipient (< 25%); critical (25–49%); unsatisfactory (50–75%); and adequate (> 75%) [35]. Additionally, the municipalities’ maximum and minimum IDs were calculated to show the range.

In addition, we measured the ID of the nine PharmSe components and of the two dimensions. The component ID was calculated as the median of the indicators in each component, considering the total sample. The same method was applied to calculate the dimension ID [10, 34]. Additionally, the ID range (minimum and maximum) was calculated to show the difference among municipalities.

## Results

The general characteristics of the 4030 PharmSes are presented in Table 1 and indicate wide geographic and demographic diversity. The study analysed 10% of the municipalities of 17 Brazilian states, corresponding to 25% of the population of those states. In absolute terms, Minas Gerais state had the largest number of municipalities included. Tocantins had the highest proportion of municipalities included, while São Paulo state had the lowest.

**Table 1:** Main characteristics of the QualiSUS-Network project sample. Brazil, 2015

State*	Municipalities	Population (Million)	Proportion of QualiSUS participants		Gini index		DHI		Pharmacies	
									Attached to health centres	Exclusive buildings
	N Total	N Total	% Mun^**2**^	% Population	Total	QualiSUS (Mean)	Total	QualiSUS (Mean)	QualiSUS (N)	
Amazonas	62	3.48	15%	6%	0.65	0.63	0.674	0.534	24	0
Bahia	417	14.02	6%	7%	0.62	0.55	0.660	0.589	211	8
Ceará	184	8.45	10%	9%	0.61	0.54	0.682	0.614	241	11
Distrito Federal	1	2.57	100%	100%	0.63	0.63	0.824	0.824	95	2
Goiás	246	6.00	7%	17%	0.55	0.50	0.735	0.685	178	8
Maranhão	217	6.57	10%	11%	0.62	0.54	0.639	0.613	146	2
Mato Grosso do Sul	78	2.45	24%	21%	0.56	0.54	0.729	0.672	118	8
Minas Gerais	853	19.60	11%	31%	0.56	0.47	0.731	0.685	406	67
Pará	143	7.58	17%	40%	0.62	0.55	0.646	0.616	277	0
Paraná	399	10.44	7%	31%	0.53	0.46	0.749	0.691	339	17
Pernambuco	185	8.80	23%	54%	0.62	0.54	0.673	0.629	721	18
Piauí	224	3.12	13%	37%	0.61	0.54	0.646	0.599	183	0
Rio de Janeiro	92	15.99	13%	62%	0.59	0.47	0.761	0.716	312	4
Rio Grande do Sul	496	10.70	4%	32%	0.54	0.42	0.746	0.730	205	15
Santa Catarina	293	6.25	8%	16%	0.49	0.43	0.774	0.736	178	0
São Paulo	645	41.26	1%	6%	0.56	0.47	0.783	0.791	144	2
Tocantins	139	1.38	41%	40%	0.6	0.53	0.699	0.631	85	5
**Total**	**5565**	**190.76**	**8%**	**22%**	**0.59**	**0.51**	**0.727**	**0.656**	**3863**	**167**

^1^States sampled represent 17 of 27

^2^Mun = Municipalities

^3^Primary health centres: primary health units/health centres/health postsSources: QualiSUS-Network data base, 2015; IBGE and IPEA, 2010

The Gini index showed that the included municipalities had a level of inequality (mean = 0.51) higher than the average of their respective states (mean = 0.59). The HDI presented the same trend. Amazonas’s sampled municipalities displayed an HDI 0.14 points lower than the average of the state, which represented the worst figure among all the sampled states.

In terms of the types of PharmSes, 95% were located within health centres. Pernambuco was the state with the highest number of PharmSes analysed, followed by Minas Gerais and Paraná.

### Municipal pharmaceutical service implementation degree

Overall, the implementation degree (ID) of PharmSes in Brazilian PHC was evaluated as critical. The ID was critical in 81% of the municipalities (*n* = 369), incipient in 14% (*n* = 65) and unsatisfactory in 4.8% (*n* = 22). The average ID within the unsatisfactory group was 53%. This shows that the ID of those municipalities is nearer to the lower limit of the group (50%). No municipality presented an adequate ID (75% or above) (Table 2).

**Table 2:** Primary care pharmaceutical services’ implementation degree in Brazilian municipalities by state, 2015

State	Municipalities by Implementation Degree								
	Incipient (< 25%)			Critical (25–49%)			Unsatisfactory (50–75%)		
	N Mun^a^	Mean %	Min-Max %	N Mun	Mean %	Min-Max %	N Mun	Mean %	Min-Max %
Amazonas	2	19	(18–20)	7	29	(27–35)	0	–	–
Bahia	1	21	21	26	32	(25–42)	0	–	–
Ceará	1	23	23	18	34	(28–38)	0	–	–
Distrito Federal	–	–	–	1	35	35	0	–	–
Goiás	1	23	23	17	33	(25–41)	0	–	–
Maranhão	10	22	(17–25)	12	32	(26–41)	0	–	–
Mato Grosso do Sul	0	–	–	17	38	(32–49)	2	54	(53–56)
Minas Gerais	10	22	(18–25)	80	36	(26–49)	8	54	(51–59)
Pará	20	19	(1–24)	4	31	(27–34)	0	–	–
Paraná	1	21	21	26	36	(26–41)	2	52	(50–53)
Pernambuco	6	24	(22–24)	37	33	(26–45)	0	–	–
Piauí	0	–	–	27	36	(26–48)	1	55	55
Rio de Janeiro	3	23	(22–24)	8	29	(26–34)	1	53	53
Rio Grande do Sul	0	–	–	16	38	(31–48)	5	53	(51–60)
Santa Catarina	0	–	–	20	40	(29–49)	2	53	(53–53)
São Paulo	0	–	–	7	36	(25–46)	0	–	–
Tocantins	10	22	(18–25)	46	33	(25–47)	1	50	50
**Total**	**65**	**22**	**(1–25)**	**369**	**34**	**(25–50)**	**22**	**53**	**(50–60)**

^a^Mun Municipalities

Regarding ID variation, the municipality presenting the worst ID was located in Pará (northern region), while that with the best was located in Rio Grande do Sul (southern region).

### Implementation degree by dimensions and components

In terms of the PharmSes components, the highest ID was achieved by forecasting (58%). In contrast, continuing education and counselling showed the lowest figure (ID = 11%) in the whole sample, followed by information and communication and teamwork. Additionally, only three components (forecasting, storage and dispensing) displayed IDs above 50%. Planning and management, which is considered a transversal component, had a critical ID (30%) (Table 3).

**Table 3:** Pharmaceutical services implementation degree by dimension and component, 2015

Dimension	Component	% Implementation degree				
		Mean (SD)	Median	Min- Max	Mean (SD)	Median
–	Planning and Management	30(16.4)	27	(0–100)	30(16.4)	27
Medicine management	Selection	21(15.2)	23	(0–73)	46(23.3)	48
	Forecasting	58(20.3)	59	(0–100)		
	Storage	54(17.9)	55	(0–100)		
	Dispensing	52(17.3)	53	(11–100)		
Care management	Pharmaceutical care coordination	28(13.9)	29	(0–70)	22(24.9)	13
	Continuing education and counselling	11(11.7)	8	(0–74)		
	Teamwork	31(40.7)	10	(0–100)		
	Information and Communication	18 (16.2)	13	(0–87)		

**Fig. 2:**
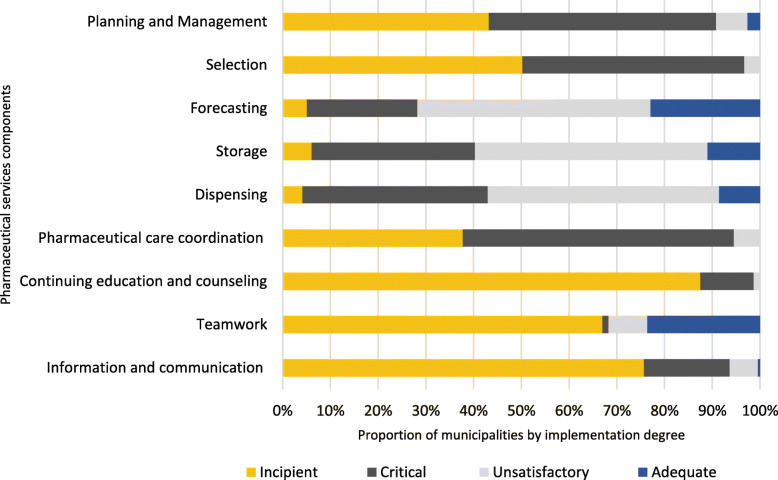
Pharmaceutical services components in Brazilian primary health care by implementation degree, 2015

Figure 2 shows the proportion of municipalities by the ID of the PharmSes component ranked by dimension. It highlights, in detail, the information presented in Table 3. The proportion of municipalities with an incipient component ID was very high. In at least 35% of the municipalities, all of the components (excluding forecasting, storage and dispensing) had an incipient ID. However, some municipalities had an adequate ID in 6 PharmSes components: planning and management (3%), forecasting (23%), storage (11%), dispensing (9%), teamwork (24%), and information and communication (1%). Teamwork reached the highest proportion among those components. Nevertheless, it also had an incipient ID in a large proportion of municipalities.

Regarding the PharmSes dimensions, medicine management (MM) ID was considered critical (Mean = 46%), while the ID of care management (CM) was incipient (Mean = 22%). In MM, all the components except selection had an ID higher than the mean of the dimension. In CM, pharmaceutical care coordination and teamwork had an ID above the mean of the dimension, indicating that those components had a better ID than that of the rest of the dimension. However, considering the median, only pharmaceutical care coordination performed above 25%. The other three components scored less than the median of the group (Table 3).

The variation between the minimum and maximum ID was extremely high regardless of the component analysed (0–100%).

## Discussion

This is the first study explicitly addressing fidelity regarding PharmSes implementation in Brazil. We used secondary data from previous research, that despite it proved to be a challenge strategy, seemed to be a good alternative in terms of saving resources and time. The sample was considered complex due to the number of municipalities, the many types of PharmSes and the variety of demographic and economic municipality characteristics.

Overall, we found that PharmSes ID in Brazilian PHC is at a critical level. The situation is even worse when we examine the PharmSes dimensions and components. The PharmSes implementation began in 1998 and is still in process. It started with the transition towards the establishment of a new municipality responsibility, set out by the National Medicines Policy (NMP). This legal arrangement transferred the duty to provide PHC medicine from the federal government to municipalities [36]. Since then, manuals and recommendations have been published to support the transition.

NMP is considered a milestone in terms of access to and the quality and adequate use of medicine in Brazil, and its priorities were built around three key elements: decentralization, funding, and logistic actions [13]. This means that most of the resources and investments have been concentrated in one PharmSe dimension - medicine management (MM). This probably explains why MM presented a mean ID two times higher than that of care management (CM) (Table 3).

There is no consensus in the literature on how long it takes to implement an intervention. It depends mainly on the intervention scale, planning, resource availability, complexity and leadership engagement [37–39]. However, considering the 15-year timeframe adopted by the Sustainable Development Goals [40] as a reasonable time to implement complex health interventions, it was expected that at least all the MM components would have reached a 75% ID.

The municipalities showed more capacity to deliver forecasting, storage and dispensing than other PharmSes components, and the improvement over the years can be identified in the literature. Recent studies presented better scenarios for inventory management [41] and dispensing [42] than studies [43, 44] from the first decade of the 21st century. However, it seems to be a consensus that those components still present many significant issues, such as lack of access to inventory software, inadequate storage conditions or lack of pharmacist supervision. These unsatisfactory conditions are fully associated with the ID of those components and medicine availability. Mendes et al. [45] found an association between a higher availability of medicine in PHC and adequate infrastructure (storage area, air conditioning, and refrigeration) and pharmacist support.

Selection drew attention due to its incipient ID. The same result was found by Margarinos Torres et al. [46] in a national qualitative study. This study evaluated standard parameters defined by the World Health Organization [47], such as published Drug and Therapeutics Committee (DTC) formal documents and the Essential Medicines Lists (EML). The authors found that only 5 of 15 municipalities visited had published their EML. Their medicine selection process involved the formal existence of a DTC. The nonexistence of published documents does not necessarily mean that the municipalities did not follow any selection criteria or did not know about the national EML. However, the existence of both EML and DTC ensures medicine quality and safety.

The results for the care management (CM) dimension showed a catastrophic scenario. All CM components had a mean ID below 31%. Pharmaceutical care coordination performed slightly better than any other component of this dimension. Considering the data collection period, these figures might be related to the formal inclusion of the pharmacist in the Family Health Program through the Family Health Support Team in 2008 [48]. This put some pressure on municipalities to consider the importance of pharmaceutical care management. Additionally, in the following years, the Pan-American Organization published the ‘Pharmaceutical Services in Primary Health Care: Position Document’ [4], and the MoH launched a series of training initiatives to increase the focus on patient care [11, 49].

### Fidelity

Clearly, the intervention has not been implemented as intended. Ninety-five percent of the municipalities had an ID < < 50%, and only 5% presented an ID above this threshold, which means low or very low implementation fidelity for most of them. General consensus indicates that 80 to 100% fidelity to the manual represents ‘high’ fidelity of delivery, whereas < 50% represents “low fidelity” [17, 50].

There is no agreement in the literature regarding whether 100% implementation is necessary to provide successful interventions. According to Barber et al. [51], an intervention has a greater chance of achieving its best performance at a moderate level of manual adherence. Interventions should have some level of adaptability, especially when they are implemented in the real world and in different contexts. The intervention can be adapted, but the essential components should be held constant.

However, poor implementation is likely to result in loss of program effectiveness. Low fidelity affects the reliability and internal validity of the intervention [52] and has a negative impact on intervention outcomes [53].

Furthermore, low fidelity is an expression of the implementers’ lack of interest in the results of the intervention [54]. Therefore, access to medicine and its adequate use seem not to be a priority as an essential right and part of the PHC.

This low PharmSes implementation is still highly relevant because it is an intervention with a specific and dedicated budget. Municipalities receive financing from the federal government to purchase PHC essential medicine. When those services display very low or low implementation fidelity, it may indicate some misuse or poor management of these resources.

Evaluating whether an intervention has been adopted with fidelity helps to explain why innovations succeed and fail. It is an opportunity to identify what has changed in a program and how these changes impact the outcomes. In addition, it reveals significant information about the feasibility of an intervention [16]. In this way, assessing fidelity can highlight training needs and aspects of program delivery that require improvement [17]. In this sense, it is clear that a set of measures should be put in place by policy makers to improve the implementation of all PharmSes components, especially those related to care management.

### Primary health care

PHC is considered an important strategy to achieve universal health coverage. It is a tool to solve approximately 80% of all health issues and to empower communities regarding their health and social condition [1]. In Brazil, PHC coverage reached 76% in October 2020 according to the MoH [55]. PHC relies on access to health products such as medicine and vaccines. Likewise, these products must be delivered in a timely manner and good condition to guarantee quality, safety, efficacy and adequate use.

Around the world, community pharmacies and PharmSes have supported PHC in delivering a range of services from prevention to management of chronic health conditions. In Brazil, regardless of whether they are within health units or in separate buildings, it is through SUS PharmSes that communities have access to PHC essential medicines [56]. Particularly for low-income families, the governmental supply is the only way to obtain medicine [57]. Essential medicines are considered by experts [47, 58, 59] to be among the most impactful health technologies in the world, and their availability, accessibility and acceptability are pivotal for many health treatments.

PharmSes have a direct impact on PHC and are considered by the WHO to be a strategic intervention [4, 59] because maintaining the dispensing and supply of several types of medicines is accompanied by the provision of many community health services, such as medicine review, counselling and support for minor ailments. Not delivering the expected activities or achieving expected outcomes means, first, no access to medicine. The literature shows that the average availability of tracer medicines in PHC in Brazil has been reported to be lower than 55% [45, 56]. This low score suggests eventual issues with components of the MM dimension.

### Inequalities

To the extent that access to medicine is still a problem in Brazil and is worse in the poorest population strata, ensuring access to medicine through the government can be considered an important strategy to reduce health inequities. Access to medicine in a timely and uninterrupted manner increases confidence in the resolvability of health issues through PHC and reduces household expenditure, which, in many cases, can account for 50% of a family’s budget [60].

The findings in this study corroborate previous findings showing that the condition of the health system and especially of PHC strategies, such as PharmSes, receive less attention in the poorest regions. The North, Northeast and Midwest regions, which according to the Brazilian Institute of Geography and Statistics (IBGE – acronym in Portuguese) are considered the poorest in the country, have the lowest prevalence of medicine use, while the South and Southeast regions have a higher prevalence [61]. Such data agree with the findings of this study, in which the municipalities in the North and Northeast presented the worst degree of implementation, while the South and Southeast stood out with better scores. Brazilian studies have shown that better socioeconomic conditions have an important correlation with higher primary health coverage [62].

Investing in proper PharmSe implementation means reducing morbidity and mortality and contributing to reducing poverty and improving living conditions.

### Strengths and limitations

This evaluation process might be used as a baseline for other studies in the future, as it evaluates the PharmSes ID based on a broad scope of activities and a range of municipalities in Brazil. This has enabled us to see the big picture in detail and deeply analyse which components need more investment.

Additionally, using the same measurement system to evaluate fidelity in different municipalities is innovative and a challenge. This study focused on examining in detail the PharmSes core components. Thus, more studies should be carried out to evaluate other aspects of fidelity and PharmSes outcomes.

The data presented in this research are the most recent at the national level, despite the data collection period. The source study is the only one in the country in which all the governmental pharmaceutical facilities in the surveyed municipalities are addressed. It was part of an important government program called QualiSUS-Network. This study constitutes a very potent baseline.

The SUS has been experiencing constraints as a result of recent political changes. We have seen health policy changes since the data collection that might indirectly affect PharmSes, such as financing regulations and program termination. However, it is exactly because of these changes that a baseline study is crucial to understanding the impact of the policy changes.

Certain limitations should be highlighted. Despite the relevance of the sample, it comprised 8.4% of the Brazilian municipalities. The methods rely on self-reporting of activities, which might be subject to reporting bias.

There are a variety of ways to measure implementation fidelity [16, 53, 63–65]. Overall, the literature suggests that 5 elements need to be considered: (a) adherence, (b) dose, (c) quality of delivery, (d) participant responsiveness and (e) program differentiation. Although there is a consensus that measuring implementation fidelity involves these 5 elements, the literature offers different ways of carrying this out. The first view suggests that all five elements need to be measured to capture a more comprehensive picture of the process [16, 53]. Second, it can be measured using either adherence or dose or quality of delivery; each of these three elements represents an alternative way to measure fidelity [64, 65]. The third view proposes the measurement of all of these elements and introduces two additional elements [63]. In the current study, we addressed one of these elements (adherence). This may have led to a limited analysis of implementation fidelity that did not capture the big picture. Other dimensions may contribute to the extent to which the intervention has been delivered.

Regarding the selection component, this study evaluated standard parameters defined by the WHO [47], such as formal documents of the DTC and the EML. Using more flexible indicators, such as the percentage of the municipal EML that is in accordance with the national EML, might lead to better representation of the state of the municipalities.

## Conclusion

The degree to which the PharmSes were implemented was critical (ID< 50%). Forecasting, storage and dispensing presented better scores, although their IDs were classified as unsatisfactory. Selection, information and communication and continuing education and counselling had incipient IDs. This analysis demonstrated that PharmSes were implemented with low fidelity, which may be related to low medicine availability in PHC, but other studies should be carried out to verify this hypothesis. Although care management components require more attention, considering their incipient ID, all components must be reviewed. Municipalities must increase their investments in PharmSes implementation to maximize these services’ benefits and guarantee the essential right of access to medicine. Further work is also required to assess the contextual factors and intervention characteristics that can affect implementation.

## Data Availability

The dataset used and analysed during the current study is available from the corresponding author on reasonable request.

## Abbreviations

PharmSes: pharmaceutical services
PHC: primary health care
ID: implementation degree
MM: medicine management
CM: care management
NMP: National Medicine Policy
NPSP: National Pharmaceutical Services Policy
CFF (Acronym in Portuguese): Federal Council of Pharmacy
ANVISA (Acronym in Portuguese): National Health Surveillance Agency
PNAUM (Acronym in Portuguese): National Research on Access and Use of Medicines
MoH: Ministry of Health
WB: World Bank
HDI: Human Development Index
DTC: Drug and Therapeutics Committee
EML: Essential Medicines List
IBGE (Acronym in Portuguese): Brazilian Institute of Geography and Statistics;
WHO: World Health Organization.
